# A Rare Case of Metallosis Co-occurring With Chronic Prosthetic Joint Infection in Total Hip Arthroplasty

**DOI:** 10.7759/cureus.21515

**Published:** 2022-01-23

**Authors:** Allicia O Imada, Brandon M Painter, Bryce Clinger, Michael M Decker

**Affiliations:** 1 Department of Orthopaedic Surgery and Rehabilitation, University of New Mexico School of Medicine, Albuquerque, USA; 2 Department of Orthopaedic Surgery and Rehabilitation, University of New Mexico Sandoval Regional Medical Center, Rio Rancho, USA

**Keywords:** two-stage revision, prosthetic joint infection, adverse local tissue reaction, metallosis, total hip arthroplasty (tha)

## Abstract

Prosthetic joint infection (PJI) and metallosis are known complications of total hip arthroplasty (THA) and are causes for revision surgeries. Articulating metal surfaces in total hip arthroplasty with corrosion at modular junctions can lead to the release of metal ions that can cause an immune-mediated biological reaction. There are few cases in the literature of both coinciding together. We describe a case of chronic *Cutibacertium acnes* PJI and metallosis co-occurring in a 64-year-old female after THA with a dual mobility construct. After undergoing uncomplicated left THA through a modified Hardinge approach, the patient dislocated anteriorly after four weeks and required revision of her acetabular component to a less anteverted position. Nine months later, she presented with hip pain and was found to have medial wall fragmentation and cystic changes around the greater trochanter on radiographs, elevated serum cobalt and chromium levels, and a benign noninfected hip aspiration. During her revision procedure, intraoperative histopathology showed over 20 neutrophils per high power field in multiple samples and fluid aspirates demonstrating Gram-positive rods. She was also found to have pseudotumor formation with the erosion of the anterior and posterior capsules with black debris on the femoral stem trunnion and the backside of the modular dual mobility liner. An antibiotic spacer was placed and her cultures grew into *C. acnes*. She completed six weeks of intravenous ceftriaxone and, during her "drug holiday," she dislocated her spacer and was found to have a lateral femoral diaphyseal stress fracture at the distal end of her spacer. She underwent stage II of her revision, and while the plan was to continue her antibiotics, she had an adverse reaction and was transitioned to oral antibiotics for six months. Due to delayed healing, she underwent additional irrigation and debridement with head and liner exchange. Her wound then healed, and at her one-year final follow-up, she was able to ambulate without pain, and her serum inflammatory and metal ion levels were within normal limits. Concurrent PJI and metallosis from articulating metal interfaces can occur, and a high index of suspicion is necessary to properly manage both conditions.

## Introduction

Metallosis is a known complication of articulating metal interfaces in total hip arthroplasty (THA) and is one type of adverse local tissue reaction (ALTR) [1]. Corrosion at modular junctions leads to the release of cobalt and chromium metal ions that penetrate soft and bony tissues, causing an immune-mediated biological reaction [2]. Patients typically present with groin, buttock, thigh, or peritrochanteric pain that is worse with weightbearing. Imaging findings include osteolysis on a radiograph as well as pseudotumor on MRI. Serum cobalt and chromium levels, erythrocyte sedimentation rate (ESR), and C-reactive protein (CRP) are often elevated. The overall incidence is as high as 5.3% in all types of THAs [3]. Treatment involves revision THA to remove the offending interfaces, diseased bone, and tissue in the periprosthetic space.

Prosthetic joint infections (PJI) occur in up to 2% of primary THAs and are the cause of approximately 15% of revisions [4]. PJI is caused by surface-adhering pathogens that create biofilms that are resistant to host defenses and microbial agents. There are various criteria for diagnosis, including sinus tract to the joint, two positive cultures of the same organism, elevated inflammatory markers, synovial cell counts, and alpha-defensins [5]. Treatment involves either partial or entire surgical removal of the prosthesis along with prolonged antibiotic therapy depending on the suspected timing and cause of infection [6].

There are a few cases in the literature of metallosis and PJI co-occurrence in total joint arthroplasty with various proposed mechanisms of metal ions leading to an increased risk of infection [7-10]. We present a rare case of a patient with early metallosis superimposed on chronic PJI (*Cutibacertium acnes*) in a THA with a modular dual mobility construct. Informed verbal consent was obtained prior to the preparation and submission of this manuscript.

## Case presentation

**Figure 1:**
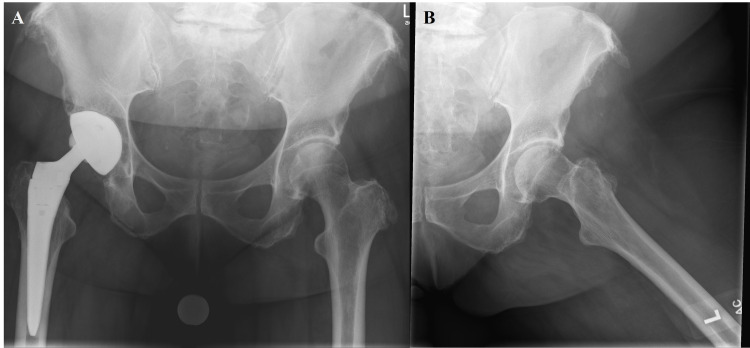
Preoperative anterior-posterior (A) and lateral (B) hip radiographs demonstrating moderate left hip osteoarthritis and a right total hip arthroplasty with a modular neck construct.

A 63-year-old female presented with two years of left hip pain and a history of a right THA 10 years prior with good results. Radiographs demonstrated moderate osteoarthritis (Figure 1A, 1B), and serum metal ions and inflammatory markers were collected due to the presence of a modular neck construct in the right THA, which were found to be within normal limits. She failed nonoperative management, including full temporary pain relief from an intraarticular corticosteroid injection, and had evidence on magnetic resonance imaging (MRI) of cartilage loss. While her radiographs did not show severe osteoarthritis, radiographs do not always correlate with pain [11,12]. The patient underwent an uncomplicated left THA (Stryker Trident, modular dual mobility, high offset stem) using a posterolateral approach. Two grams of cefazolin were given after the induction of anesthesia, prior to skin incision. She was discharged on postoperative day (POD) 2 with a negative pressure incisional wound vac dressing (Avelle, ConvaTec, Deeside) as is standard in our practice.

**Figure 2:**
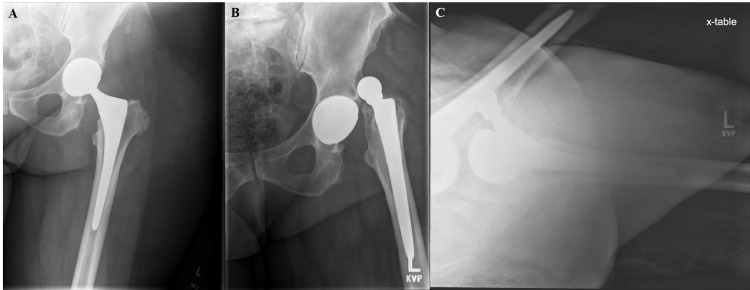
Anterior-posterior hip radiograph (A) at two weeks postoperatively after undergoing left THA (Stryker Trident, modular dual mobility, high offset stem) using a modified Hardinge approach. Patient sustained a fall and dislocated anteriorly as seen on anterior-posterior (B) and cross table lateral (C) radiograph.

**Figure 3:**
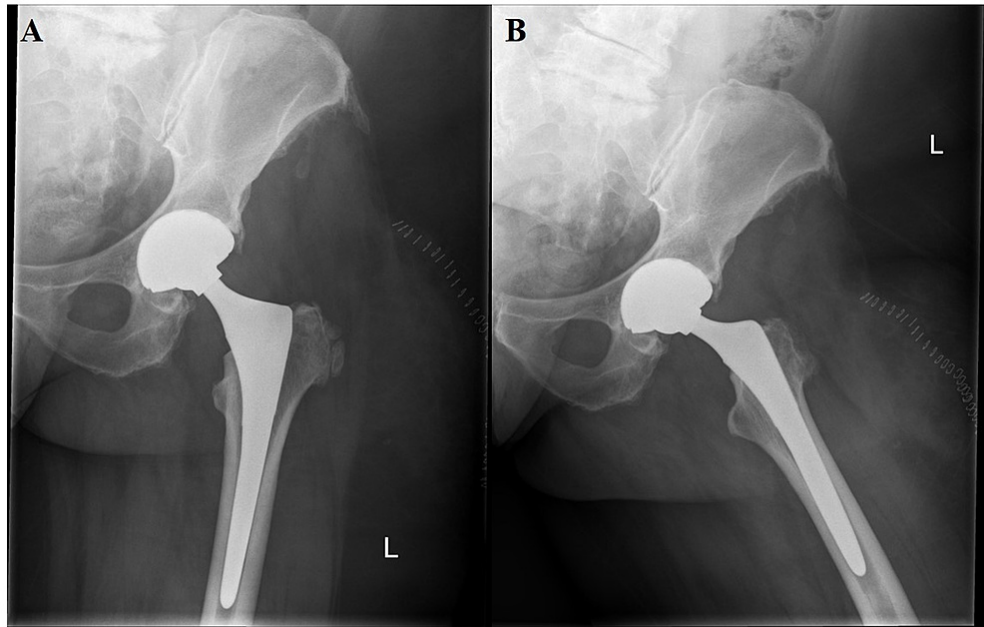
Two-week postoperative anterior-posterior (A) and lateral (B) radiographs after first revision surgery to a larger head and cup with decreased anteversion.

The patient developed early postoperative superficial wound breakdown, requiring frequent wound checks, dry dressing changes, and a short course of prophylactic oral antibiotics. The wound did heal and her staples were removed at POD 11 (Figure 2A). Her inflammatory labs were decreasing as expected. Her C-reactive protein decreased from 4.3 mg/dl (ref <0.3 mg/dl) on post-operative day 6 to 0.6 mg/dl at four weeks. Unfortunately, at four weeks postoperatively, the patient stepped awkwardly and sustained an anterior dislocation of her THA (Figure 2B, 2C). Radiographic appearance was concerning for intraprosthetic dislocation, which was a contraindication for closed reduction, so the decision was made to bring her to the operating room. Intraoperatively, we found that the bipolar head had not dissociated but was dislocated anteriorly. However, the version of the acetabular component was determined to be inappropriate for this patient, and the decision was made to revise her to decrease anteversion to reduce her future risk of dislocation (Figure 3A, 3B). Intraoperative cultures were negative. Her postoperative course went as expected, without wound complications and with normal radiographs. At five months, postoperative radiographs demonstrated a lucency medial to the acetabular component.

**Figure 4:**
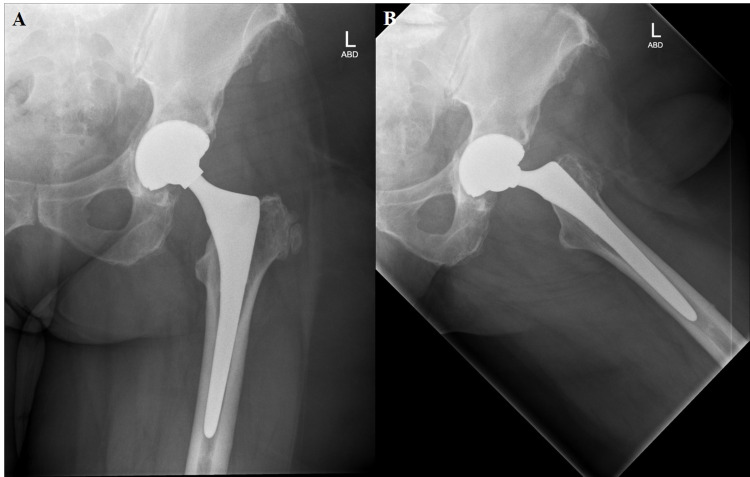
Nine-month postoperative anterior-posterior (A) and lateral (B) radiographs with signs of cup loosening. There is medial wall fragmentation and cystic change around the greater trochanter.

At nine months, she presented to the clinic with one week of increasing hip pain. She had pain with passive hip range of motion. Imaging showed previously noted medial wall fragmentation and cystic changes around the greater trochanter (Figure 4A, 4B). ESR was 91 mm/hour and CRP was 6.7 mg/dL, increased from 14 mm/hour and 0.6 mg/dL, respectively. Metal ions were also drawn with cobalt 4.4 µg/L (ref ≤3.9 µg/L) and chromium 1.7 µg/L (ref ≤5.0 µg/L). Hip aspiration showed cloudy, amber-colored fluid containing 1583 total nucleated cells, 7% neutrophils, 59% lymphocytes, and 34% mononucleated cells. Alpha defensin (Synovasure) was negative.

**Figure 5:**
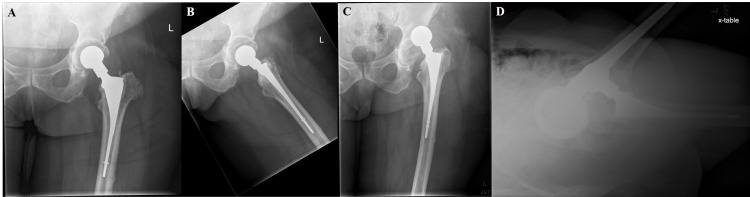
Six-week postoperative anterior-posterior (A) and lateral (B) radiographs after stage 1 of second revision procedure with placement of Zimmer antibiotic spacer. One week later, patient stepped awkwardly and dislocated the spacer (anterior-posterior - C, cross table lateral - D).

Given these findings, the patient was scheduled for revision THA. Intraoperative histopathology confirmed over 20 neutrophils per high power field in multiple samples and fluid aspirates demonstrating Gram-positive rods. There was also pseudotumor formation with the erosion of the anterior and posterior capsules with black debris on the femoral stem trunnion and the backside of the modular dual mobility liner. A thorough debridement was completed and a stage 1 revision with an antibiotic spacer was implanted with vancomycin and tobramycin cement (Figure 5A, 5B). She was made to touch down, weight-bearing. Cultures grew *C. acnes* and she was discharged on ceftriaxone 2 g intravenous daily and rifampin 300 mg twice a day for six weeks per infectious disease (ID) recommendations. Her wound healed appropriately. Antibiotics were discontinued after six weeks when her ESR and CRP normalized to 5 mm/hour and 0.3 mg/dL, respectively. The plan was for a two-and-a-half-week antibiotic holiday, rechecking her labs, and proceeding, if appropriate, with stage 2 of her revision.

The patient, unfortunately, stepped awkwardly the following week and dislocated her spacer (Figure 5C, 5D) and had evidence on imaging of a lateral femoral diaphyseal stress fracture at the distal end of her spacer. After discussion with the patient about the residual risk of infection given that it had not been the full antibiotic holiday timeframe versus the morbidity of an extra surgical procedure, her second stage revision was completed the following day. Intraoperative histopathology and cultures were found to be negative. Despite this, ID recommended continuing ceftriaxone and rifampin due to concern about residual infection. The patient had an adverse reaction to ceftriaxone with flushing, pruritis, paresthesias, muscular weakness, palpitations, chest pain, and dyspnea. Therefore, she was transitioned to cephalexin at 1 g every eight hours and then to 500 mg for a total of six months.

On POD 16, she developed wound drainage and was taken to the operating room for irrigation and debridement, head and liner exchange, and wound vacuum placement. She was discharged on POD 3 with an ID recommendation to continue her current antibiotic regimen.

**Figure 6:**
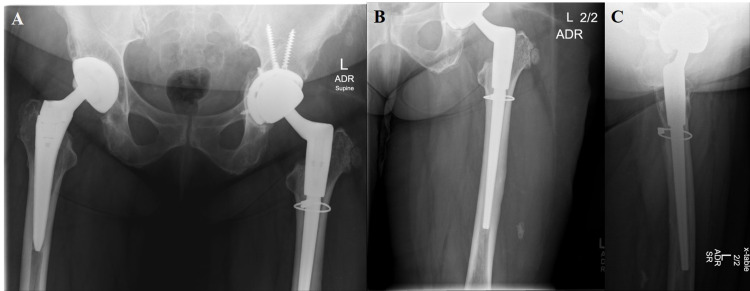
One-year postoperative radiographs [anterior-posterior pelvis (A), anterior-posterior left hip (B), lateral left hip (C)] after stage 2 of second revision using Stryker Res Mod system and cabling for femoral neck stress fracture with successful eradication of infection.

Her wound healed, and she progressed with physical therapy. At a one-year follow-up, she has weaned off all ambulatory aids and has no pain (Figure 6A-6C), with normal serum metal ions (cobalt <10.0 µg/L, chromium 1.6 µg/L) and inflammatory markers (ESR 17 mm/hour, CRP 1.0 mg/dL).

## Discussion

To the best of our knowledge, we report the first case of metallosis and chronic PJI with isolated *C. acnes* in THA. PJI and metallosis do not often co-occur, with only case reports in the current literature [7-10]. These case reports describe nine patients: three THAs, three total knee arthroplasties, and three Birmingham hip resurfacings treated at various institutions with explants, one-stage revisions, or two-stage revisions and antibiotics. Infectious agents include *Klebsiella pneumonia* and various species of Streptococcus, Staphylococcus, and Enterobacter. One case involved multiple bacteria, including two streptococcus species, *C. acnes*, and staph hominis [7]. The patient in our study had an infected revision THA after an initial revision for instability at nine months postoperatively. Infection was confirmed intraoperatively with over 20 neutrophils per high powered field, and while her aspiration numbers were not conclusive, they had grown Gram-negative rods by the time her stage one surgery occurred.

In previous case reports, the diagnosis was suspected preoperatively due to imaging (osteolysis, pseudotumor), serology (cobalt, chromium, ESR, CRP), aspiration, intraoperative findings, and/or histology. All these patients presented with pain and difficulty bearing weight, as in our case. Intraoperatively, evidence of ALTR includes metal-stained fluid/soft tissue, pseudocapsule, and pseudotumor [1]. In our patient, we found erosion of the anterior and posterior capsules with black corrosion of the femoral stem trunnion and modular metal acetabular liner intraoperatively. Histology can be an important diagnostic tool and will usually show necrotic and hemorrhagic tissues with a mixed inflammatory infiltrate of lymphocytes, plasma cells, and macrophages with intracytoplasmic metal debris [7,9].

Treatment and outcome varied greatly in the various case reports. Multiple patients were treated successfully with one-stage revision and intravenous antibiotics, while others required multiple irrigations and debridements, and one reported death. We propose that the bacteria’s virulence, as well as the chronicity of actual infection, may play a role here. Our patient required multiple procedures but is not on life-long antibiotic suppression as in previous case reports. *C. acnes* has been found to be implicated in 4-6% of PJIs and has a high rate of successful eradication due to antibiotic susceptibility [13,14].

There is some evidence that metallosis may increase the risk of infection in total joint arthroplasty. Prieto et al. found a higher rate of infection in their MoM THAs (DePuy ASR) at the Mayo Clinic at 5.6% PJI compared to their normal documented 1.3% [15]. The exact cause of this is not clear. There is evidence that metal debris causes the release of proinflammatory cytokines [16]. Anwar et al. demonstrated that any type of particulate debris provides a scaffold for bacteria and biofilm formation, while cobalt and chromium debris specifically accelerate bacterial growth in vitro [17]. Our patient, unfortunately, had a very long and complicated clinical course due to her infection, metallosis, and instability. It is impossible to determine if PJI or metallosis occurred first, but there was some radiographic evidence of medial wall lucency at five months postoperatively.

## Conclusions

While we often work to diagnose PJI or metallosis as our differential for painful THA, we need to keep in mind that they co-occur. Our case demonstrates the successful diagnosis and treatment of one such patient. Her clinical course was long and complicated, but at one year postoperatively, she was able to ambulate with a nonpainful hip.

Metallosis and PJI can present together in total hip arthroplasty and must not be missed to ensure appropriate treatment. These can be successfully treated with both antibiotics and staged revision surgery.
